# Growth Promotion of Rice and *Arabidopsis thaliana* by Volatile Organic Compounds Produced by Endophytic *Clonostachys* Species

**DOI:** 10.3390/jof10110754

**Published:** 2024-10-30

**Authors:** Hui Chen, Jin Xu, Dengke Shao, Chunfang Zhao, Xiaohong Xu, Xihui Xu, Chen Chen

**Affiliations:** 1 College of Life Sciences, Nanjing Agricultural University, Nanjing 210095, China; 9201010120@stu.njau.edu.cn (H.C.); 2021116106@stu.njau.edu.cn (J.X.); 2024216015@stu.njau.edu.cn (D.S.); xiaohong.xu@njau.edu.cn (X.X.); 2 Institute of Food Crops, Jiangsu Academy of Agricultural Science, Nanjing 210014, China; czhao@jaas.ac.cn; 3 Jiangsu Collaborative Innovation Centre for Solid Organic Waste Resource Utilization, Nanjing Agricultural University, Nanjing 210095, China

**Keywords:** plant–endophytic fungi, volatile organic compounds, plant growth-promoting

## Abstract

Plant–endophytic fungi are widely distributed and highly diverse, with many of them capable of influencing plant growth and development, which is related to the production of volatile organic compounds (VOCs). While certain fungal VOCs have been found to stimulate plant growth, others exhibit inhibitory effects. Importantly, the impact of fungal VOCs extends beyond host plants to affect non-host plants as well. In this study, we isolated two plant–endophytic fungi, *Clonostachys* sp. CC1 and *Clonostachys* sp. CC2, from healthy rice roots. These strains were co-cultured with both rice and *Arabidopsis thaliana*. Our results demonstrated that both strains significantly enhanced the growth of both rice and *A. thaliana*. Specifically, they increased the length and biomass of rice and *A. thaliana* seedlings, as well as the chlorophyll content, while decreasing the H_2_O_2_ content in the leaves of both plants. The VOCs produced by these strains were analyzed using gas chromatography–mass spectrometry (GC-MS), which identified a total of 10 main ingredients. Among these compounds, 1-pentanol, ethylbenzene, and dimethyl phthalate inhibited the growth of rice while promoting the growth of *A. thaliana*, highlighting the variability in the effects of these compounds on different plant species and the complexity of plant–fungal interactions.

## 1. Introduction

Plant–endophytic fungi refer to a group of fungi that live inside the tissues and organs of plants at a certain stage or all stages without affecting the hosts negatively [1]. Many plant–endophytic fungi play an important role in plant growth, which is related to the volatile organic compounds (VOCs) they produce. VOCs, characterized by their low molecular weight and ability to evaporate readily, serve as vital communication tools in interspecies interactions [2]. VOCs produced by the plant–endophytic fungi have been reported to be able to promote plant growth and improve plant adaptability to various biotic and abiotic stresses by facilitating nutrient absorption, modulating hormone levels, and inducing systemic resistance. For example, *Trichoderma* sp. produces growth hormone-like substances, such as cytokinins, which have been shown to enhance seedling emergence rates and increase the height and leaf area of plants like tomato, cucumber, and chili; *Trichoderma viride* has been observed to stimulate *Arabidopsis* growth, promote lateral root formation, and induce early flowering, even without direct physical contact [3,4]. Additionally, research by Kuswinanti et al. highlights the potential of endophytic fungi from aromatic rice, which produce growth-promoting substances like indole-3-acetic acid (IAA) and lignin for rice growth, as well as organic acids for phosphorus solubilization [5]. These fungi have shown higher efficiency in phosphorus solubilization compared to bacteria.

Studies have also shown that VOCs produced by some plant–endophytic fungi could inhibit plant growth [6,7,8]. The possible reasons were that these compounds disrupt essential ion channels or decrease enzyme activity crucial for plant growth and development [6]. For example, VOCs from *Tuber melanosporum*, such as 3-Octenol and (E)-2-Octenal, inhibit *A. thaliana* root and leaf growth, accelerate leaf oxidation, and increase ROS scavenging enzyme activity [7]. Similarly, VOCs from *Aspergillus flavus* and *Fusarium oxysporum* reduce the length of barley roots by 19 to 21.5% and the surface of *Hordeum vulgare* aerial parts by 15% [8]. Furthermore, different concentrations of fungal VOCs can have varying effects on plant growth, suggesting a dose-dependent relationship. For instance, 6-Pentyl-2H-pyran-2-one (6-PP) from *Trichoderma* sp. promotes *Arabidopsis* growth at lower concentrations but inhibits it at higher concentrations [9]. Likewise, 1-hexanol, a common VOC produced by bacteria and fungi, promotes plant growth at low concentrations but inhibits it at high concentrations [10]. Therefore, understanding the effect of these VOCs on plant growth is crucial for the application of VOCs.

In this study, we isolated two plant–endophytic fungi, *Clonostachys* sp. CC1 and *Clonostachys* sp. CC2, from the healthy roots of rice. To date, several secondary metabolites from *Clonostachys* spp. have been identified, including terpenes, polyketides, and peptides [11]. These compounds exhibit antagonistic effects against bacteria, inhibit the formation of phytopathogenic fungal adherent cells, and promote plant growth. For instance, Xiao et al. screened out *Clonostachys rosea* from medicinal plants, which significantly promoted the growth of *Anoectochilus roxburghii*, an endangered Chinese medicinal herb [12].

Despite extensive research on *Clonostachys* spp., there has been limited investigation into the specific components of their VOCs and their effects on plant growth and development. Therefore, this study aims to address this gap by employing gas chromatography–mass spectrometry (GC-MS) to analyze the specific components of the VOCs produced by *Clonostachys* sp. CC1 and *Clonostachys* sp. CC2. Furthermore, we measured the influence of individual compounds among the VOCs on plant growth and development. Our findings reveal that VOCs produced by these two strains can promote the growth of both rice and *Arabidopsis*. Notably, we identified VOCs that inhibited the growth of rice while promoting the growth of *A. thaliana*, highlighting the variability in the effects of these compounds on different plant species. Specifically, we identified 1-pentanol, ethylbenzene, and dimethyl phthalate as potential fumigants for promoting the growth of *A. thaliana*, paving the way for the application of VOCs produced by *Clonostachys* species.

## 2. Materials and Methods

### 2.1. Fungal Strains

Fungal strains CC1 and CC2, which were isolated from rice roots, were used in this study. Both strains were cultured in a PDA medium (Haibo Biotechnology Co., Ltd., Qingdao, China) at 26 °C. The genomic DNA of the two strains was extracted separately with an E.Z.N.A^®^ Fungal DNA mini kit following the manufacturer’s guidelines (Omega, Darmstadt, Germany). To identify the two strains, ITS genes were amplified by PCR following the method of Jing et al. [13]. The maximum likelihood (ML) tree implemented in MEGA X [14], the Tamura-Nei model, and 1000 bootstrap replicates were used to perform phylogenetic analysis.

### 2.2. Co-Culture of Fungi and Plants

Exposures of rice and *A. thaliana* plants to *Clonostachys* VOCs were performed using a double plate-within-a-plate system to avoid direct contact with plants and fungal strains. Briefly, a small, uncovered Petri dish (35 × 35 mm; Solarbio Science and Technology Co., Ltd., Beijing, China) containing *Clonostachys* grown on PDA was placed into a larger partitioned Petri dish (150 × 150 mm) containing two (rice) or eight (*A. thaliana*) plant seedlings.

Healthy, plump seeds of rice and *A. thaliana* were selected for the co-cultivation of plants and fungi. Seeds of rice were surface-sterilized by soaking in 75% ethanol solution (Shandong Lilkang Medical Technology Co., Ltd., Dezhou, China) for 1 min and NaClO solution (0.9% available chlorine content; Sinopharm Chemical Reagent Co., Ltd., Shanghai, China) for 30 min, and then rinsed with sterile deionized water 8 times. The rice seeds were placed on Petri dishes (100 × 100 mm) containing half-strength Murashige and Skoog salt (1/2 MS medium; Solarbio Science and Technology Co., Ltd., Beijing, China) in a plant growth chamber. The seeds were kept in dark for 2 days to promote root growth and then cultured under a 16 h light/8 h dark photoperiod at 26/24 °C with a relative humidity of 70% and light intensity of 10,000 lm/m^2^. Similarly, the seeds of *A. thaliana* were surface-sterilized by soaking in 75% ethanol solution for 20 s and NaClO solution (3% available chlorine content) for 10 min and then rinsed with sterile deionized water 8 times. The seeds were first kept in the refrigerator at 4 °C for 2 days and then cultured in a light incubator with the same condition as rice.

For inoculation of fungal strains, a plug (5 mm diameter) from a PDA plate containing the fungus was placed in the small Petri dish containing PDA medium. The PDA culture medium without strains was used as the control group. The rice and *A. thaliana* seedlings with uniform growth after 7 days of cultivation were transferred to a large Petri dish containing 1/2 MS medium, with 2 seedlings for *A. thaliana* and 8 seedlings for rice per Petri dish. Each treatment included 5 biological replicates. The fungi and plants were co-cultured in a plant growth chamber at 26/24 °C with a 16 h light/8 h dark photoperiod. At 7 days after inoculation, plant samples were harvested to measure the shoot height, root length, fresh weight, hydrogen peroxide content, and photosynthetic pigment content of each sample. Each treatment included 5 biological replicates.

### 2.3. Determination of Hydrogen Peroxide and Chlorophyll Content in Plant Tissues

To measure hydrogen peroxide contents in plant tissues, 0.2 g of fresh samples were added to 2 mL of 0.1% TCA, grinded thoroughly on ice, and centrifuged at 1200× *g* for 15 min. The supernatant (0.5 mL) was mixed with 0.5 mL of PBS (10 mmol/L, pH = 7.0) and 1 mL of KI, and then the absorbance was measured at the wavelength of 390 nm.

The contents of chlorophyll a, chlorophyll b, and carotenoids were determined according to the method of Lichtenhaler and Wellburn [15]. Briefly, 0.1 g of plant leaves were cut into pieces, added to 10 mL of ethanol–acetone (1:1) mixture, and kept in dark overnight (not more than 12 h). The absorbance was measured at the wavelengths of 663, 646, and 470 nm when the leaves turned white. The following formulas were used to calculate the content of each chlorophyll component:

C_a_ = 12.21 × A_663_ − 2.81 × A_646_ (1)

C_b_ = 20.13 × A_646_ − 5.03 × A_663_ (2)

C_x−c_ = (1000 × A_470_ − 2.05 × C_a_ − 114.8 × C_b_)/245 (3)

### 2.4. Identification of VOCs Produced by the Two Clonostachys Strains

For headspace volatile analysis, 5 mL of PDA slant medium was injected into a headspace flask with a capacity of 20 mL, placed obliquely, inoculated the strains after solidification, and incubated at 26 °C for 10 days. *Clonostachys* cultures were grown in a 20 mL glass flask (Zhejiang Sainz Scientific Instrument Co., Ltd., Ningbo, China) containing 5 mL of PDA at 26 °C for 10 days. Headspace samples taken from sterile PDA served as negative controls. The headspace was collected for 1 h and analyzed by GC-MS. The VOCs were extracted by a solid phase microextraction (SPME; Agilent Technologies Co., Ltd., California, America) device, and an SPME fiber (50/30 μm Divinyl benzene/Carboxen/Polydimethylsiloxane, DVB/CAR/PDX; Anpel Laboratory Technologies Co., Ltd., Shanghai, China) was used to absorb VOCs. The VOCs were analyzed using a gas chromatograph–mass spectrometer (Agilent 7890N/5975C system; Agilent Technologies Co., Ltd., California, America) equipped with an HP-5MS quartz capillary column (0.25 mm × 30 m, 0.25 μm). The detection conditions of the column were as follows: the extraction time was 45 min, the carrier gas was helium, the column flow rate was 1.0 mL/min, the temperature of the vaporization chamber was 240 °C, the column temperature was maintained at 30 °C for 3 min, then the temperature was increased to 220 °C at a rate of 5 °C/min and then maintained at 270 °C for 1 min after the operation, and the injection volume was 1.0 μL in a non-split flow mode. The mass spectrometry conditions were as follows: the ionization voltage was 70 eV, the EI ionization source and ion source temperature was at 230 °C, the mass spectral range was 20~450 amu, and the mass spectral scanning rate was 3.35 scans/s. The VOCs were identified by comparison with the NIST/EPA/NIH Mass Spectral Library 2005 (NIST05) database (Agilent).

### 2.5. In Vitro Growth Promotion of Arabidopsis thaliana and Rice Seedlings by Pure VOCs with Different Concentrations

The capacities of the pure 1-pentanol, ethylbenzene, and dimethyl phthalate for the promotion of the growth of rice and *A. thaliana* were evaluated in vitro. These VOCs were previously identified in the cultures of the tested *Clonostachys* strains by GC-MS. The experiment was performed in Petri dishes, as described above for fungi, but by replacing the *Clonostachys* inoculum with filter papers that were impregnated with 10 μL aqueous solutions of the pure VOCs. These solutions included standard solutions and those diluted 10×, 100×, and 1000×. The standard solution consisted of 99.5% 1-pentanol (concentration in vials: 0.018 mg/mL) (Macklin, Shanghai, China), 99.5% ethylbenzene (concentration in vials: 0.019 mg/mL) (Macklin, Shanghai, China), and 99.7% dimethyl phthalate (concentration in vials: 0.026 mg/mL) (Macklin, Shanghai, China). The Petri dishes that contained seedlings and filter papers without VOCs were used as the control group. After 7 days of culture, the plant-growth-promotion parameters were measured as indicated above. Each treatment included 5 biological replicates.

### 2.6. Data Analysis

IBM SPSS Statistics 21 was used for statistical analysis. The results were subjected to an analysis of variance (ANOVA) with Duncan test and Dunnett’s *t*-test (*p* < 0.05). The Origin 2021 was used for graphing.

## 3. Results

### 3.1. Characterization of Two Clonostachys Isolates

**Figure 1:**
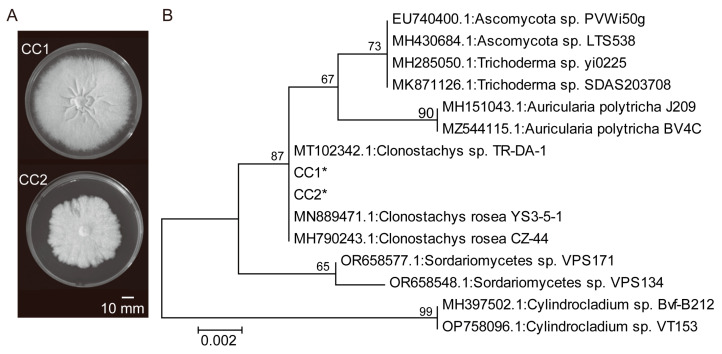
Identification of strains CC1 and CC2. (**A**) Colony morphology of the two strains. (**B**) Phylogenetic relationships between the two strains and related species. The maximum likelihood (ML) phylogenetic tree is shown. The ML bootstrap values based on 1000 replications are indicated above the branches. Asterisks indicate the strains isolated in this study.

Two fungal strains, CC1 and CC2, were isolated from healthy rice roots (Figure 1). Both strains exhibited growth on the PDA medium, and strain CC1 grew faster than strain CC2 (Figure 1A). The ML tree based on ITS sequences revealed that strains CC1 and CC2 clustered with *Clonostachys rosea*, supported by a bootstrap value of 87% (Figure 1B). These findings indicate that both strains can be assigned to the genus *Clonostachys*.

### 3.2. Impacts of VOC Mixture Produced by the Two Clonostachys Strains on the Growth and Development of Rice

**Figure 2:**
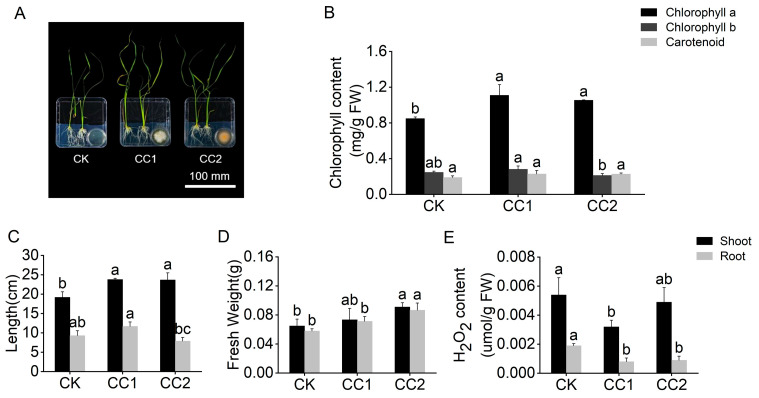
Impacts of VOCs produced by strains CC1 and CC2 on the growth of rice. (**A**) Promotion of rice growth by the two strains at 7 days after inoculation. (**B**) Chlorophyll content in rice leaves. (**C**) Length of rice shoots and roots. (**D**) Fresh weight of rice shoots and roots. (**E**) H_2_O_2_ content of rice shoots and roots. The letters a, b, and c in the figure represent the results of Duncan’s multiple range test (*p* < 0.05), which is used to indicate the significance of differences between groups. Letters that do not overlap between two groups indicate a significant difference between those groups.

To investigate the effects of VOCs produced by *Clonostachys* strains on the growth and development of rice, 5-day-old rice seedlings with uniform growth were co-cultured with 2-day-old strains for 7 days to assess rice growth under VOC exposure (Figure 2A). Compared with the control group, rice exposed to VOCs exhibited a significant increase in shoot height and fresh weight (Figure 2B,C). For instance, VOCs produced by *Clonostachys* sp. CC2 led to a 65.33% increase in the fresh weight of rice shoots. Additionally, the VOCs significantly promoted root growth, as revealed by an increase in the number of lateral roots and root fresh weight (Figure 2C,D). These findings demonstrate the significant growth-promoting effects of *Clonostachys* VOCs on rice.

To explore the impact of VOC exposure on the photosynthetic capacity of rice leaves, the chlorophyll content was measured (Figure 2B). VOCs produced by both strains significantly increased rice chlorophyll content, particularly chlorophyll a (Figure 2B). Furthermore, to assess the effect of VOCs on rice oxidative activity, H_2_O_2_ contents in shoot and root tissues of rice exposed to VOCs for 7 days were analyzed. Under VOC treatment, there was a significant decrease in H_2_O_2_ content in both shoot and root tissues (Figure 2E). For example, VOCs from *Clonostachys* sp. CC1 reduced H_2_O_2_ content in rice shoots and roots by 38.46% and 57.89%, respectively. These results indicate that VOCs produced by the two *Clonostachys* strains can mitigate H_2_O_2_ accumulation in rice leaves and roots, thereby promoting rice growth and development.

### 3.3. Impacts of VOC Mixture Produced by the Two Clonostachys Strains on the Growth and Development of Arabidopsis

**Figure 3:**
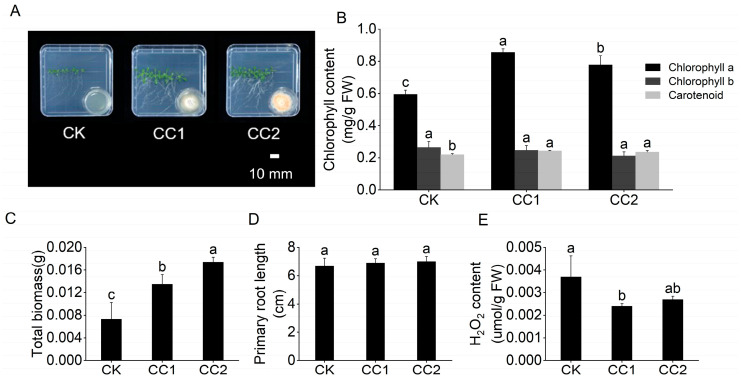
Impacts of VOCs produced by strains CC1 and CC2 on the growth of *Arabidopsis thaliana*. (**A**) Promotion of *A. thaliana* growth by the two strains at 7 days after inoculation. (**B**) Chlorophyll content in *A. thaliana* leaves. (**C**) Total biomass of *A. thaliana*. (**D**) Length of *A. thaliana* roots. (**E**) H_2_O_2_ content of *A. thaliana* shoots. The letters a, b, and c in the figure represent the results of Duncan’s multiple range test (*p* < 0.05), which is used to indicate the significance of differences between groups. Letters that do not overlap between two groups indicate a significant difference between those groups.

To further explore the impact of VOCs on different plant species, we utilized the model plant *A. thaliana*. Similar to rice, *Clonostachys* VOCs significantly enhanced *A. thaliana* growth after 7 days of co-cultivation (Figure 3). Specifically, VOCs produced by strain CC2 increased the total fresh weight of *A. thaliana* by 138.36% (Figure 3C). Additionally, *Clonostachys* VOCs markedly reduced the H_2_O_2_ content in *A. thaliana* leaves (Figure 3E). Moreover, *Clonostachys* VOCs significantly augmented the chlorophyll content of *A. thaliana* leaves, with strain CC1’s VOCs elevating chlorophyll a and carotenoid content by 43.7% and 10.59%, respectively (Figure 3B). These findings demonstrate the comparable growth-promoting effects of *Clonostachys* VOCs on both rice and *A. thaliana*.

### 3.4. Identification of VOC Mixture Produced by the Two Clonostachys Strains

The two *Clonostachys* strains were cultivated separately on PDA for 7 days, followed by the collection of headspace samples over a 4 h period for GC-MS analysis. Variances in types and abundance of volatiles were observed between the two *Clonostachys* strains. A total of 10 volatile compounds were identified, including alcohols, aldehydes, alkenes, esters, and aromatic compounds (Table 1). Among these, five volatile compounds were found to be common to both strains, such as 2-methyl-1-butanol, 2-isopropyl-5-methyl-2-hexenal, dimethyl phthalate, and 2,6,10-trimethyltetradecane (Table 1).

### 3.5. Effects of Pure VOCs of Different Concentrations on the Growth of Rice and A. thaliana Seedlings

**Figure 4:**
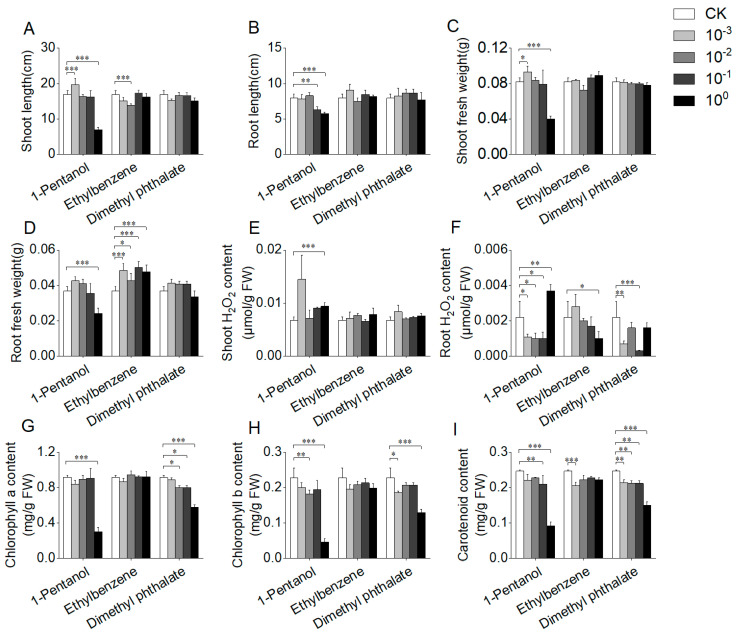
Impact of pure compounds on the growth of rice. The phenotypic changes of rice after 7 days of cultivation with the three pure compounds. (**A**) Shoot length of rice. (**B**) Root length of rice. (**C**) Fresh weight of rice shoots. (**D**) Fresh weight of rice roots. (**E**) H_2_O_2_ content of rice shoots and roots. (**F**) H_2_O_2_ content of rice roots. (**G**) Chlorophyll a content in rice leaves. (**H**) Chlorophyll b content in rice leaves. (**I**) Carotenoid content in rice leaves. Four different concentrations of pure compound were used, including standard solutions (10^0^) and those diluted 10× (10^−1^), 100× (10^−2^), and 1000× (10^−3^). The “*”, “**”, and “***” in the figure represent the result of Dunnett’s *t*-test (*p* < 0.05, 0.01, and 0.001, respectively), indicating the significant difference between treatment and control.

**Figure 5:**
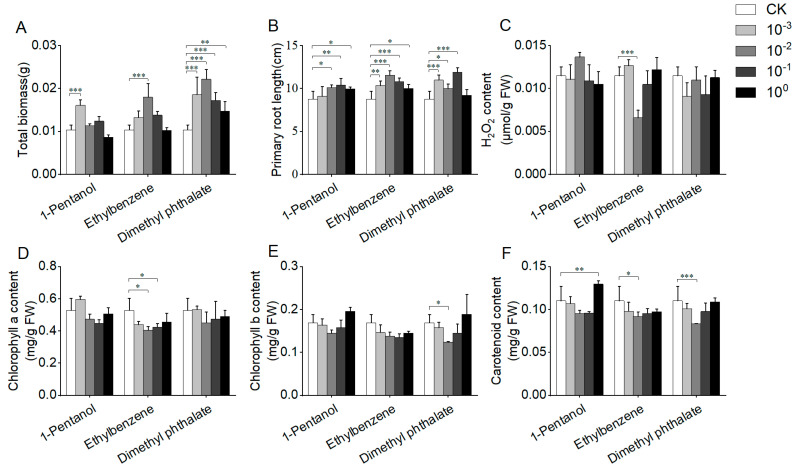
Impact of pure compounds on the growth of *Arabidopsis thaliana*. The phenotypic changes of *A. thaliana* after 7 days of cultivation with the three pure compounds. (**A**) Total biomass of *A. thaliana*. (**B**) Primary root length of *A. thaliana*. (**C**) H_2_O_2_ content of *A. thaliana*. (**D**) Chlorophyll a content in *A. thaliana* leaves. (**E**) Chlorophyll b content in *A. thaliana* leaves. (**F**) Carotenoid content in *A. thaliana* leaves. Four different concentrations of pure compound were used, including standard solutions (10^0^) and those diluted 10× (10^−1^), 100× (10^−2^), and 1000× (10^−3^). The “*”, “**”, and “***” in the figure represent the result of Dunnett’s *t*-test (*p* < 0.05, 0.01, and 0.001, respectively), indicating the significant difference between treatment and control.

Out of the 10 detected VOCs, three pure compounds (1-pentanol, ethylbenzene, and dimethyl phthalate) were procured and utilized to investigate their effects on plant growth (Figure 4 and Figure 5). In rice, 1-pentanol exhibited strong growth inhibition, and the standard solution of 1-pentanol resulted in reductions of shoot length, root length, shoot fresh weight, and root fresh weight by 59.15%, 27.2%, 51.58%, and 34.24%, respectively (Figure 4A–D). Additionally, 1-pentanol significantly decreased the chlorophyll a, chlorophyll b, and carotenoid contents in rice by 67.03%, 79.67%, and 62.85%, respectively, while also elevating the H_2_O_2_ content in rice leaves (Figure 4E–I). Notably, the inhibition of rice growth was progressively alleviated as the concentration of 1-pentanol was reduced, with lower concentrations of 1-pentanol promoting rice growth (Figure 4A–D). In contrast, the standard solutions of ethylbenzene and dimethyl phthalate showed relatively weak effects on rice growth compared to 1-pentanol. Specifically, weak changes in shoot and root length and fresh weight were observed in rice treated with ethylbenzene and dimethyl phthalate compared with controls (Figure 4A–D). Moreover, decreased carotenoid contents were detected in treatments with ethylbenzene and dimethyl phthalate (Figure 4I). Diluted solutions of ethylbenzene and dimethyl phthalate exhibited effects on rice growth similar to those of the standard solutions. These findings suggest that ethylbenzene and dimethyl phthalate are not the active compounds in *Clonostachys* VOCs contributing to rice growth promotion.

In contrast to rice, different effects of the tested VOCs on *A. thaliana* seedlings were observed (Figure 5). Although the standard solutions of 1-pentanol, ethylbenzene, and dimethyl phthalate showed weak effects on the growth of *A. thaliana*, diluted solutions exhibited significant promoting effects on *A. thaliana* growth (Figure 5A,B). For instance, the total biomass and primary root length of *A. thaliana* increased by 19.23% and 18.68% when treated with 1000× dilution of 1-pentanol (concentration in vials: 0.000018 mg/mL). In addition, the standard solution of 1-pentanol resulted in the increase of chlorophyll a, chlorophyll b, and carotenoid contents of *A. thaliana* and weak changes in the H_2_O_2_ content (Figure 5C–F). Additionally, both diluted solutions of ethylbenzene and dimethyl phthalate decreased the H_2_O_2_ content of *A. thaliana* significantly (Figure 5C). These results showed the growth-promoting activity of 1-pentanol, ethylbenzene, and dimethyl phthalate on *A. thaliana*.

## 4. Discussion

The interactions between plant endophytic fungi and plants are of great interest. Endophytic fungi residing within plant tissues contribute to plant growth through the production of various secondary metabolites and enzymes, thereby exhibiting diverse biological activities. For example, their influence on plant growth is notably mediated by VOCs they emit during their growth phases. Minerdi et al. [16] demonstrated that bicyclic semiterpenes released by *Aspergillus flavus* FS2 and *Fusarium oxysporum* MSA-35 promoted the growth of lettuce seedlings and increased the expression of expansion protein genes in the leaves significantly [17]. Additionally, *F. oxysporum* was found to enhance shoot and root growth in *Arabidopsis* and tobacco, and the process was governed by plant growth hormone signaling pathways [17]. Furthermore, VOCs emitted by *Phoma* sp. GS8-3, categorized as plant growth-promoting fungi (PGPF), encompassed compounds like 2-methylpropanol, 3-methylbutanol, methacrylic acid, and isobutyl acetate [18]. These compounds exhibited substantial growth-promoting effects on tobacco in vitro [18]. Interestingly, their effectiveness was more pronounced at lower concentrations, highlighting a dose-dependent relationship. While these VOCs resulted in a modest increase in tobacco fresh weight, their efficacy in promoting growth underscores their potential utility in agricultural practices [18]. Notably, the endophytic fungi also release a certain amount of CO_2_ during interactions between fungi and plants, which may not necessarily promote plant growth. For example, in a study comparing *Anoectochilus formosanus* grown in a closed environment, those inoculated with *Tolypocladium* sp. did not show significant differences in fresh weight, average number of roots, or shoots compared to control plants that were not inoculated with mycelium [19]. This suggests that the CO_2_ released during the interaction may not have a notable growth-promoting effect on these plants.

The *Clonostachys* strains, such as *C. rosea*, are filamentous fungus that exhibits a wide distribution worldwide [20]. It holds significant importance in various fields, including bioenergy, biodegradation, biological fermentation, and biological control [20,21]. Furthermore, *C. rosea* has been shown to promote plant growth and development. For instance, it facilitates the germination of pepper and eggplant seeds while also increasing the height of seedlings [22]. *C. rosea* can also promote the growth of tomato plants [21,23,24] and boost root development in lettuce grown hydroponically [25]. In this study, we observed significant growth promotion effects of two plant endophytic fungi, *Clonostachys* sp. CC1 and *Clonostachys* sp. CC2, isolated from the inter-root soil of healthy rice. Both strains exhibited noticeable enhancements in the growth parameters of rice and *A. thaliana*. Specifically, they induced a vibrant green coloration in rice leaves, increased shoot thickness, elevated plant height, and enhanced fresh weight of both shoot and root. Similarly, *Arabidopsis* plants treated with these strains displayed greener leaves, expanded leaf surface area, and augmented total biomass. Additionally, both strains led to an increase in chlorophyll content within the leaves of both rice and *A. thaliana*. Therefore, the two strains could be prioritized as promising candidates for biofumigant applications aimed at enhancing plant growth.

Hydrogen peroxide (H_2_O_2_) is one of the primary representatives of reactive oxygen species (ROS), and its production and accumulation in plant cells can be triggered by biotic and abiotic stresses, as well as hormonal signals [26,27,28,29,30]. The VOCs emitted by both CC1 and CC2 strains caused a general reduction in H_2_O_2_ content within rice and *A. thaliana* tissues. This suggests a potential role for these VOCs in directly or indirectly scavenging ROS, thereby benefiting plant growth and development. Notably, fungal VOCs have been demonstrated to affect plant hormone signaling pathways [31], leading to acidification of the cell wall, which in turn loosens the cell wall and promotes cell elongation [32]. These results suggested the complex interactions of VOCs, ROS, and plant growth.

Different concentrations of pure VOC compounds have varying effects on plant growth. For example, high concentrations (10 and 100 mg/L) of mushroom alcohol (1-octen-3-ol) effectively inhibited the germination of *A. thaliana* seeds and the growth of their seedlings [33]. Similar inhibitory effects were observed for 1-hexanol [10]. To further investigate the effects of pure compounds produced by strains CC1 and CC2 on the growth of rice and *A. thaliana* at different concentrations, a comparative analysis was conducted. Compared to the control group, the greatest increase in the total biomass of *A. thaliana* was observed with a 1000× dilution of 1-pentanol (concentration in vials: 0.000018 mg/mL) and a 100× dilution of ethylbenzene and dimethyl phthalate (concentrations in vials: 0.00019 and 0.00026 mg/mL, respectively). While the total biomass of *A. thaliana* increased, the chlorophyll content in the leaves did not show a significant increase, suggesting that these compounds did not enhance plant growth by boosting leaf photosynthesis. These results suggest that pure VOCs could serve as potential growth-promoting fumigants for *A. thaliana*. In contrast, the effects on rice were different. Although exposure to the standard solution of 1-pentanol decreased the biomass and chlorophyll content of rice leaves, dilutions of 1-pentanol not only alleviated these inhibitory effects but also promoted rice growth. Ethylbenzene and dimethyl phthalate exhibited relatively weak inhibitory effects on rice growth compared to 1-pentanol, and their diluted forms had similar effects. These findings indicate that the tested pure VOCs, especially 1-pentanol, inhibited the growth of rice but promoted the growth of *A. thaliana*. The concentration of these VOCs significantly influenced their growth-promoting effects, highlighting the variability in their effects on different plant species. Additionally, *Candida nivariensis* has been shown to produce 1-pentanol [34], whereas no endophytic fungi have been reported to produce ethylbenzene and dimethyl phthalate to date.

Although the two *Clonostachys* strains, CC1 and CC2, were derived from the same host and isolation site, they exhibited different production of VOCs, providing evidence for individual differences between homologous strains. It is important to note that due to experimental limitations, we obtained only three pure VOCs (1-pentanol, ethylbenzene, and dimethyl phthalate). Consequently, we conducted growth promotion experiments on rice and *A. thaliana* using different concentrations of these pure compounds. However, it is also possible that untested compounds possess plant growth-promoting activity. Moreover, the observed bioactivities of VOCs, such as growth promotion, may result from the synergistic effects of multiple compounds. For example, *Cladosporium cladosporioides* was reported to significantly improve the growth of tobacco seedlings in vitro, and the strain produced α-pinene, (+)-sativene, dehydroaromadendrene, (-)-trans-caryophyllene, and tetrahydro-2,2,5,5-tetramethylfuran, so it was hypothesized that these five compounds may have some synergistic effect [35]. Furthermore, both VOCs (2-methyl-butanal and 3-methyl-butanal) produced by *Cladosporium halotolerans* regulated the growth and development of *Nicotiana benthamiana*, and the mixture of the two VOCs also enhanced plant growth and root development compared to the compounds alone, suggesting a synergistic effect of the mixture of the two VOCs on plant growth [36]. Furthermore, methyl jasmonate (MJ) released into the soil in a volatile form stimulates the formation of bacterial biofilms away from the plant roots. These biofilms release a different array of volatile compounds that significantly contribute to plant growth in synergy with MJ [37]. However, it is important to recognize that a single compound may not fully replicate all the bioactivities exhibited by VOCs. Therefore, further studies are required to explore the synergistic effects of different VOCs on plant growth.

## Figures and Tables

**Table 1:** Volatile compounds identified by GC-MS from *Clonostachys* VOCs.

ID	Residence Time (min)	Compound Name	*Clonostachys* sp. CC1	*Clonostachys* sp. CC2	CAS Number
C1	2.33	1,3-Pentadiene	+	−	000504-60-9
C2	5.15	2-Methyl-1-butanol	+	+	000137-32-6
C3	5.4	1-Pentanol	−	+	000071-41-0
C4	8.92	Ethylbenzene	+	−	000100-41-4
C5	18.04	Ethyl benzoate	+	−	000093-89-0
C6	20.38	8-Methyl-1-decene	−	+	061142-79-8
C7	21.71	2-Isopropyl-5-methyl-2-hexenal	+	+	035158-25-9
C8	23.73	Dimethyl phthalate	+	+	000131-11-3
C9	24.19	2,6,10-Trimethyltetradecane	+	+	014905-56-7
C10	25.67	3,4-Diethylbiphenyl	−	+	061141-66-0

## Data Availability

The original contributions presented in the study are included in the article, further inquiries can be directed to the corresponding authors.
